# Traditional Chinese Medicine ShenZhuGuanXin Granules Mitigate Cardiac Dysfunction and Promote Myocardium Angiogenesis in Myocardial Infarction Rats by Upregulating PECAM-1/CD31 and VEGF Expression

**DOI:** 10.1155/2017/5261729

**Published:** 2017-07-03

**Authors:** Dan-Ping Xu, De-Zhi Zou, Hui-Liang Qiu, Huan-Lin Wu

**Affiliations:** ^1^Cardiovascular Department, Guangdong Provincial Hospital of Traditional Chinese Medicine, Guangdong Provincial Academy of Chinese Medical Sciences, Guangzhou 510006, China; ^2^Emergency Department, The First Affiliated Hospital of Sun Yat-sen University, Guangzhou 510006, China; ^3^Second Clinical Medical College, Guangzhou University of Chinese Medicine, Guangzhou 510080, China

## Abstract

**Background:**

Myocardial infarction (MI) is the main cause of global mortality and morbidity despite the development of therapeutic approaches. ShenZhuGuanXin granules (SG) have been shown to possess cardioprotective effects against coronary heart disease (CHD). However, little is known about its specific mechanism. The present study aimed to investigate the therapeutic effect of SG in cardiac dysfunction and to demonstrate whether SG can promote myocardium angiogenesis by establishing a rat model of myocardial infarction with left anterior descending ligating.

**Methods and Results:**

Three days after MI, rats were randomly divided into six groups: sham group (sham), MI group (MI), MI + low dose SG (SG-L) group, MI + middle dose SG (SG-M) group, MI + high dose SG (SG-H) group, and MI + compound Danshen dropping pills (CDDP) group as a positive control. Four weeks after administration, rats underwent hemodynamics and echocardiography study. Ventricle tissues were processed for histology and immunohistochemistry studies. Compared with MI group, SG treatment dose-dependently improved cardiac hemodynamic function, attenuated infarct size, increased microvessel density, and increased the expression of PECAM-1/CD31 and VEGF.

**Conclusions:**

SG dose-dependently improved cardiac hemodynamic function and attenuated infarct size by promoting angiogenesis through upregulating PECAM-1/CD31 and VEGF expression.

## 1. Introduction

Studies surrounding the prognosis of myocardial infarction (MI) had become one of the most heated topics in the field of life sciences. Although the mortality of MI is significantly lower than it used to be with the development of therapeutic approaches [1–3], there remains the need for further deep study on related factors that contributed to prognosis of myocardial infarction and novel therapeutic agents. Coronary revascularization (including thrombolytic therapy, percutaneous coronary intervention, and coronary artery bypass surgery) can effectively rebuild epicardial coronary blood flow, but the objectively existing phenomenons of recurrent angina pectoris and progressive functional deterioration in patients after surgery and myocardial ischemic symptoms even in patients without coronary stenosis indicate that it does not represent the reopening of myocardial microvessel and successful myocardial reperfusion and can not completely eliminate the dysfunction of coronary microcirculation, which plays an important role in myocardial blood supply and directly affects cardiac function and metabolism [4–6].

In recent years, China has expended great efforts in developing and inheriting Tradition Chinese Medicine (TCM) for treatment of coronary heart disease (CHD). ShenZhuGuanXin granules (SG) are a Chinese patent medicine developed from ShenzhuGuanxin Recipe, an empirical prescription of Professor Deng (master of TCM) and Professor Wu [7–9]. It is composed of seven Chinese medicines, including Radix Ginseng, Radix Panacis quinquefolii, Radix Notoginseng, Hirudo, Rhizoma Pinelliae, Rhizoma Atractylodis, Folium Nelumbinis, and has been shown to possess extensive pharmacological effects on CHD such as stable angina by its prominent potential of “enhancing qi, eliminating phlegm, and accelerating the blood circulation” according to TCM theory [7, 9, 10] and clinical trials [7–9].

Although the results from a prospective, randomized controlled trial showed that SG was effective and safe to improve angina pectoris score, TCM symptom score, and Seattle Angina Questionnaire and decrease adverse events in patients with angina pectoris after percutaneous coronary intervention [8], little is known about its specific mechanism. In this experiment, we hypothesized that SG could improve cardiac function and promote myocardium angiogenesis in myocardial infarction rats by upregulating VEGF. The aims were therefore to investigate the therapeutic potential role of SG in cardiac dysfunction in rat model of MI and to demonstrate whether SG can promote myocardium angiogenesis by analyzing capillary density, PECAM-1/CD31, and VEGF protein expression.

## 2. Methods

### 2.1. Experimental Animal

Experiment protocol was approved and monitored by Animal Care Committee of Guangdong Provincial Hospital of Chinese Medicine. All procedures were performed according to Regulations of Experimental Animal Administrations promulgated by the State Committee of Science and Technology of China. A total of sixty male Sprague Dawley rats in SPF grade, weighing 200–220 grams, were obtained from Guangdong Provincial Medicinal Laboratory Animal Center (SCXK (Yue) 2013-0002, Guangzhou, China) and raised in animal experiment center of Guangdong Provincial Academy of Chinese medicine. Rats were fed with regular chow and housed in plastic cages under a normal night-day rhythm and standard laboratory control of temperature and humidity during all experiments.

### 2.2. Preparation of ShenZhuGuanXin Granules Solution

**Figure 1:**
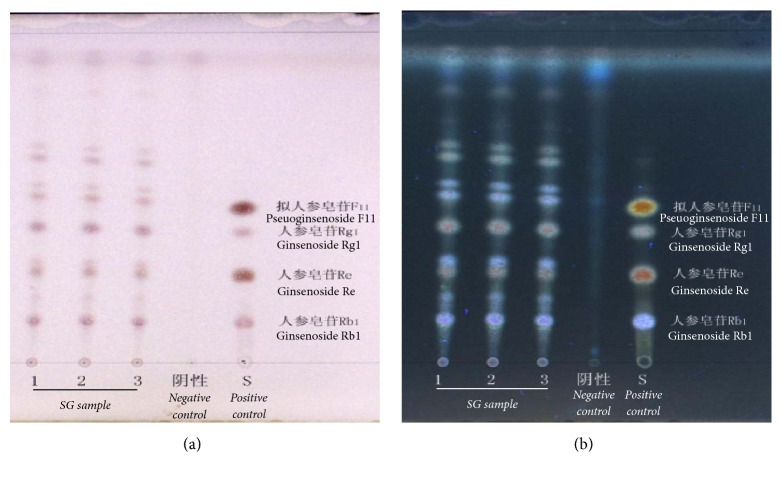
Thin-layer chromatography (TLC) was used for qualitative analysis part of active ingredients of SG, which was detected containing pseuoginsenoside F11, ginsenoside Rg1, ginsenoside Re, and ginsenoside Rb1. Solvent was used as negative control. Positive control is a mixture solution, containing four standard substances: pseuoginsenoside F11, ginsenoside Rg1, ginsenoside Re, and ginsenoside Rb1. (a) and (b) were inspected under fluorescence and ultraviolet, respectively.

**Figure 2:**
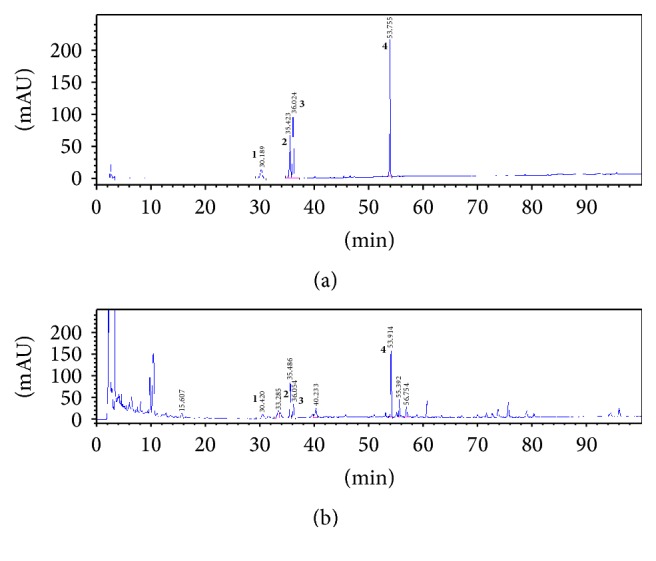
High performance liquid chromatography (HPLC) was selected for identification of the major chemical compounds of SG. Fingerprint chromatogram (a) is the peaks of four standards. Same peaks were identified in (b) (SG sample).** 1**, notoginsenoside R1;** 2**, ginsenoside Rg1;** 3**, ginsenoside Re;** 4**, ginsenoside Rb1.

ShenZhuGuanXin granules (SG) were manufactured and supplied by Jiangyin Pharmaceutical Group Co., Ltd., and mainly used in Guangdong provincial hospital of Chinese medicine for IHD, including CHD. Besides, the fingerprint of SG was analyzed by thin-layer chromatography and high performance liquid chromatography (HPLC) and the chromatograms were shown in Figures 1 and 2. SG were dissolved in aseptic distilled water at the dose of 3981.6 mg/kg (high dose SG), 1260 mg/kg (middle dose SG), and 630 mg/kg (low dose SG) for this study. In this study, compound Danshen dropping pills (CDDP) purchased from Tasly Pharmaceutical Group Co., Ltd., were chosen as a positive control and were ground and dissolved in aseptic distilled water at dose of 28.5 mg/kg.

### 2.3. Myocardial Infarction

After three days' quarantine inspection, MI model was established by ligating the left anterior descending branch of coronary artery (LAD) at the segment of two millimeters below the lower edge of left auricle. Briefly, rats received 10% chloral hydrate (250 mg/kg weight) intraperitoneal anesthesia and intubated with a polyethylene tube (16 G size) for artificial respiration (Harvard Apparatus, Model Inspira, US). After a thoracotomy was performed, LAD was ligated by using a 7-0 polyamide suture. The suture was tightened enough to block the blood flow completely. Sham operated rats underwent the same procedure, but without ligating LAD. And then, chest was closed. Three days later, echocardiography was used to screen for MI rats.

### 2.4. Animal Grouping and Experimental Procedure

MI rats were randomly divided into five groups: (1) untreated MI control group (MI); (2) MI + low dose SG (SG-L); (3) MI + middle dose SG (SG-M); (4) MI + high dose SG (SG-H); (5) MI + CDDP (CDDP). Sham operated rats were grouped into sham group (sham). 4-week course of oral administration (once a day) was commenced 3 days after MI modeling. On follow-up, all rats underwent hemodynamics analysis and echocardiography study. Ventricle tissues were processed for histology and immunohistochemistry studies.

### 2.5. Hemodynamics Analysis

A polyethylene cannula connected to pressure transducer was placed in the right carotid artery to record blood pressure and mean arterial pressure (MAP), and then the cannula was pushed into the left ventricle to record heart rate (HR), left ventricular systolic pressure (LVSP), left ventricular end-diastolic pressure (LVEDP), and maximum positive or negative first derivative of left ventricular pressure (±*dP/dt*.max) by using a multichannel physiological data acquisition system (BIOPAC Systems Inc., US).

And then, the cannula was removed. Median sternotomy was performed to better exposure ascending aortic root for assessing cardiac output (CO) by using an Ultrasound Flowmeter (Transonic 400-Series Flowmeter, Transonic Systems Inc., US) equipped with a transonic 1.5 PSL flowprobe. All of values above were at least recorded for 5 cardiac cycles and expressed as an average value.

### 2.6. Histological and Immunohistochemistry Studies

Ventricular tissue samples were separated, fixed in 10% neutral buffered formalin, sectioned (3 *μ*m thick), and stained with Masson's trichrome or hematoxylin and eosin (HE). Myocardial infarction size was measured as calculating the ratio of the average scar circumference from all sections to the average left ventricular circumference from all sections. Microvessel density (MVD) in the peri-infarct (border zone) region was calculated from 5 random views and expressed as myocardial microvessel count/mm^2^ (MVC/mm^2^).

For further analysis of capillary density, immunohistochemical analysis of platelet endothelial cell adhesion molecule-1 (PECAM-1/CD31, SC-1506, 1 : 200, Santa Cruz Biotechnology, CA, USA) was performed. Transverse sections of the short axis at the level of papillary muscle were chosen in this analysis. Five fields in the border zone at 200x magnification were randomly selected for analyzing integral optical density (IOD) value using Image Pro Plus 6.0. Likewise, we analyzed the expression of vascular endothelial growth factor (VEGF, sc-7269, 1 : 50, Santa Cruz Biotechnology, CA, USA).

### 2.7. Statistical Analysis

All values were expressed as means ± standard deviation. SPSS13.0 software was applied to analyze the values. Group differences were performed by one-way ANOVA followed by LSD test. *P* < 0.05 was considered to have a statistically significant difference.

## 3. Results

### 3.1. SG Improved Cardiac Hemodynamic Function Dose-Dependently

To investigate the therapeutic potential role of SG in cardiac dysfunction in rat model of MI, hemodynamics analysis was conducted after four weeks of medication. As shown in Table 1, MI caused a significant decrease in MAP when compared with the sham group (97.72 ± 6.00 versus 42.75 ± 6.29 mmHg, *P* < 0.05) and SG-M and SG-H treatment increased the MAP to 75.38 ± 10.25 and 78.87 ± 4.01 mmHg, respectively (*P* < 0.05). LVSP and +*dP/dt*.max are important indexes for evaluating left ventricular systolic function. MI caused a significant decrease in LVSP and +*dP/dt*.max when compared with the sham group (LVSP: 116.83 ± 9.00 versus 89.80 ± 6.76 mmHg, *P* < 0.05; +*dP/dt*.max 2724.27 ± 321.84 versus 1331.23 ± 116.49 mmHg/s, *P* < 0.05). However, SG-M and SG-H treatment significantly increased the LVSP to 101.70 ± 7.22 and 107.54 ± 2.18 (*P* < 0.05) and increased the +*dP/dt*.max to 2057.06 ± 182.27 and 2127.66 ± 129.62 (*P* < 0.05), respectively.

Besides, LVEDP and −*dP/dt*.max are important indexes for evaluating left ventricular diastolic function. LVEDP was significantly increased in MI group compared with the sham group (2.87 ± 2.91 versus 28.51 ± 5.77 mmHg, *P* < 0.05). SG-M treatment significantly attenuated the increase of LVEDP to 9.52 ± 6.13 mmHg. −*dP/dt*.max was significantly decreased in MI group compared with the sham group (2600.40 ± 300.88 versus 1319.31 ± 121.05 mmHg/s, *P* < 0.05); however, SG-M and SG-H treatment significantly attenuated this fall to 1985.16 ± 174.86 and 2046.29 ± 114.83 mmHg/s (*P* < 0.05). No significant difference was found in hemodynamic variables between MI and CDDP treatment group. In this experiment, CO was measured by a Ultrasound Flowmeter. MI resulted in a significant decrease in CO when compared with the sham group (34.67 ± 3.22 versus 14.06 ± 1.43 ml/min, *P* < 0.05), whereas SG-H treatment significantly attenuated this fall to 31.24 ± 1.98 ml/min (*P* < 0.05).

Taken together, these results indicated that SG exerts a protective effect on cardiac hemodynamic function dose-dependently against MI, and its protective effect at median and high dose is superior to CDDP at the dose of 28.5 mg/kg.

### 3.2. SG Attenuated Infarction Size Dose-Dependently

**Figure 3:**
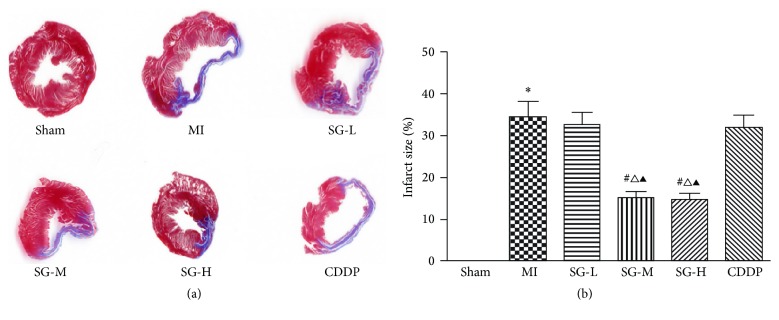
Infarction size assessment by Masson's trichrome staining. (a) Representative myocardial sections are shown. (b) SG-M and SG-H treatment significantly reduce the infarction size compared with MI group. Each value represents the mean ± SD of nine rats. ^*∗*^*P* < 0.05 versus sham group, ^#^*P* < 0.05 versus MI group, ^∆^*P* < 0.05 versus DSDW group, ^▲^*P* < 0.05 versus SG-L group.

Masson' s trichrome was used to measure the infarction size. As shown in Figure 3, the MI group exhibited a larger infarction size, thinner ventricle wall, less cardiomyocytes count, and structurally disordered distribution of collagen fibers in the infarction area when compared with drugs-treated groups. In particular, SG-M and SG-H treatment significantly reduce the infarction size to 15.07% ± 1.50% and 14.58% ± 1.46% at a dose-dependent manner when compared with MI group (34.29% ± 3.86%, *P* < 0.05). Additionally, compared with MI group, it seems that SG treatments reveal decreased collagen fibers with more horizontal and parallel distribution either in infarction zone or in border zone (data not shown).

### 3.3. SG Increased Microvessel Density Dose-Dependently

**Figure 4:**
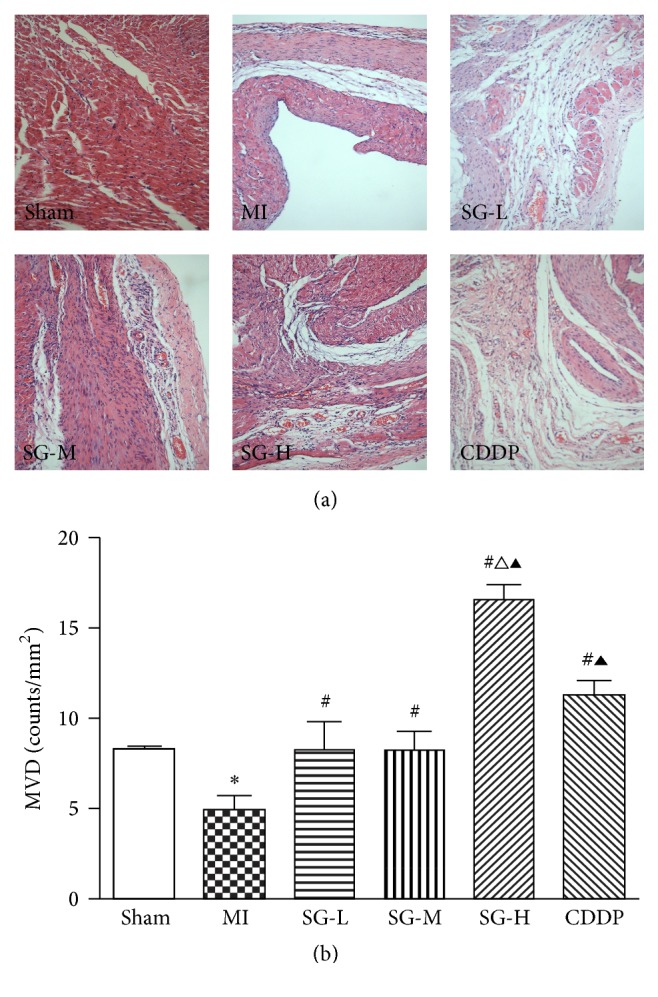
SG and CDDP treatment increased microvessel density (MVD) after MI. (a) MVD in the myocardial infarction border zone by HE staining; (b) quantitative analysis. Each value represents the mean ± SD of nine rats. ^*∗*^*P* < 0.05 versus sham group, ^#^*P* < 0.05 versus MI group, ^∆^*P* < 0.05 versus DSDW group, ^▲^*P* < 0.05 versus SG-L and SG-M group. Magnification was 200x.

As shown in Figure 4, nascent cardiac microvessels were observed in the infarction border zone by HE staining. Compared with sham group, higher capillary density was observed in MI and therapeutic groups. Compared with MI group, SG-L, SG-M, SG-H, and CDDP treatment significantly increased capillary density (*P* < 0.05). The effect of SG-H on density increment was better than CDDP (*P* < 0.05). And not only that, the effect of SG-H on density increment was more than threefold over MI group and twofold over SG-L and SG-M group.

### 3.4. SG Increased the Expression of PECAM-1/CD31 and VEGF Dose-Dependently

**Figure 5:**
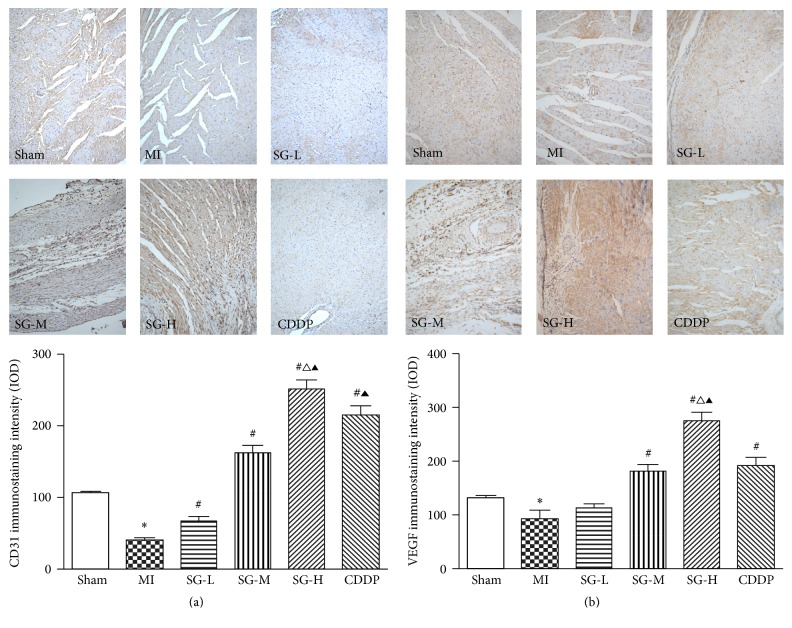
Immunohistochemical staining of PECAM-1/CD31 (a) and VEGF (b). (a) The expression of PECAM-1/CD31 was increased significantly in SG-L, SG-M, SG-H, and CDDP group compared with MI group; besides, (b) SG-M, SG-H, and CDDP treatment significantly increased the expression of VEGF compared with MI group. Each value represents the mean ± SD of nine rats. ^*∗*^*P* < 0.05 versus sham group, ^#^*P* < 0.05 versus MI group, ^∆^*P* < 0.05 versus DSDW group, ^▲^*P* < 0.05 versus SG-L and SG-M group. Magnification was 200x.

PECAM-1, a surface marker of vascular endothelial cell, also was involved in the regulation of angiogenesis and was detected by immunohistochemical staining. As shown in Figure 5(a), compared with MI group, optical intensity value of CD31, which means the expressed level of CD31, was increased significantly in SG-L, SG-M, and SG-H group in dose-dependent manner and CDDP group (*P* < 0.05). Besides, the results showed that the effects of interventions on CD31 expression in descending order were SG-H > CDDP > SG-M > SG-L, and difference between each two groups had a statistical significance.

VEGF, one of the potent angiogenic factors, was detected by immunohistochemical staining in this experiment. As shown in Figure 5(b), SG-M, SG-H, and CDDP treatment significantly increased the IOD value of VEGF when compared with MI group (*P* < 0.05). What is more, SG-H treatment showed a higher IOD value of VEGF than SG-M and CDDP group (*P* < 0.05). These results suggested that SG-M, SG-H, and CDDP treatment significantly upregulated the protein expression level of VEGF. The effect of SG was dose-dependent.

## 4. Discussion

TCM has accumulated rich experience in the treatment of CHD in the course of development for thousands of years [11, 12]. The TCM theory of “Regulating Spleen and Nourishing Heart” (RSNH) for CHD treatment developed from “five-viscera correlation theory” and proposed by a TCM master Professor Deng [13] has been used in the prevention and treatment of CHD for decades in South China [10, 14]. Since qi-deficiency, turbid-phlegm, and blood stasis were the main syndromes of patients with CHD in South China proved by a clinical and epidemiological investigation [15], RSNH theory was proposed and SG was therefore designed to “supplement qi, eliminate phlegm, promote blood circulation, and remove blood stasis” and to prevent and treat CHD. From a case report in 1982 [10] to several prospective clinical studies in recent years [7–9], it has been proved that RSNH theory and SG are really of theoretical and practical feasibility.

SG are composed of seven Chinese medicines as described [7] and numbers of potential active constituents, such as notoginsenoside R1, ginsenoside Rb1, Re, Rg1, and pseuoginsenoside F11 as shown in Figures 1 and 2. Although clinical studies have confirmed that SG are efficient in the treatment of CHD with stable angina pectoris [7–9], information on the molecular mechanisms of SG and its effect in animal model of canine MI are unknown. This study proved for the first time that SG effectively improved cardiac hemodynamic function. Possible mechanisms may be related to its function of angiogenesis. Previous studies have proved the angiogenic effect of notoginsenoside R1 [16], ginsenoside Re [17, 18], and Rg1 [17, 19], which may explain the main active constituents of SG but that is not our aim to further clarify the role of these constituents of the present study.

A small but significant proportion of patients can not really benefit or gain few benefits from PCI while no-reflow phenomenon continuously exists and remains unsolved [6]. No-reflow phenomenon is clinically related to decreased myocardial salvage index, extension of infarction size, and an increase of 5-year mortality [20]. So far, however, there is no effective intervention measure to fully ameliorate no-flow phenomenon, of which the primary cause is that coronary artery microcirculation disturbance has been gradually realized [21]. Treatment strategies aiming at improvement of microcirculation will absolutely offer the effective strategy to further improve prognosis of myocardial infarction [22]. Angiogenesis is an important repair process of the progression of ischemic heart disease (IHD) and is expected to improve the recovery of blood flow of infarction zone during MI process [23]. Therapeutic angiogenesis provides fresh leads in the quest for treatment of IHD, especially CHD, and plays a positive role in collateral vessels formation and improvement of coronary microcirculation and heart function [23, 24].

In this study, by counting capillary density directly, a basic method to access neovascularization [25], the results were found that SG-L, SG-M, and SG-H treatment significantly increased capillary density in infarction border zone at a dose-dependent manner, suggesting that SG can play a role in angiogenesis. The increased capillary density is closely associated with collateral vessels formation and coronary microcirculation [23], accounting for the effect of SG on cardiac hemodynamic improvement and reduced infarction size. Further study was focused on how SG exerted angiogenesis function. PECAM-1/CD31 and VEGF were then investigated.

Platelet endothelial cell adhesion molecule-1 (PECAM-1/CD31), a kind of cell adhesion molecules, mainly expressed on endothelial cells, circulatory platelets, monocytes, neutrophilic granulocytes, and some of subgroup of T cells, is a member of immunoglobulin superfamily, which plays a vital function in mediating cell adhesion, regulating leukocytes transmigration and platelet function, inhibiting cell apoptosis, and mediating signal transduction pathway [26]. Since PECAM-1 was mainly expressed on cell membrane of both developing and matured vascular endothelial cells, it was always considered as a marker of vascular endothelial cells. In fact, the role of PECAM-1 is essential for neovascularization [27, 28]. Stable connection between endothelial cells firstly starts by cell adhesion mediated by PECAM-1, and then endothelial cells can proliferate, migrate to perivascular matrix, and reestablish cell-cell contact, so as to develop new blood vessels [29]. Besides, intracellular signaling pathways participating in cell adhesive mechanisms also need the participation of PECAM-1 [28]. Inhibition the function of PECAM-1 by its antibody [30, 31] or by its antagonist [32] may inhibit angiogenesis.

Vascular endothelial growth factor (VEGF) is one of the most important angiogenic growth factors associated with blood vessel growth [24, 33]. By promoting the mitosis of vascular endothelial cells, VEGF plays a vital role in inducing angiogenesis and collateral vessels formation [23], which plays a critical role in IHD [33]. In the study of occlusive vascular disease, such as MI, administration of exogenous VEGF can effectively promote neovascularization and collateral vessels formation in response to tissue ischemia [34]. Targeted VEGF therapy immediately following MI may offer more additional advantages to save more myocardium by improving collateral circulation and microvascular function and decreasing infarction size, resulting in significant improvement of heart function [34, 35]. On the contrary, downregulation of VEGF will absolutely inhibit its function of angiogenesis [36, 37].

In this study, the expression of both PECAM-1 and VEGF was significantly increased by SG treatment, suggesting that SG can possess the function of angiogenesis against chronic MI. Besides, compound Danshen dripping pills (CDDP), composed of Radix* Salvia miltiorrhiza*, Radix Notoginseng, and Borneolum, a Chinese patent medicine which is enrolled into phase III clinical trial of US in 2013, are widely used for treatment of CHD. Previous clinic studies have demonstrated that CDDP is safe and effective in the treatment of CHD; its antianginal effect is even more effective than isosorbide dinitrate [38]. Animal experiment showed that CDDP can exert a protective effect against myocardial ischemia-reperfusion-induced microcirculatory disturbances [39]. Therefore, CDDP was chosen as a positive control medicine in this study. We found that CDDP exerted notable benefits on improving angiogenesis by upregulating the expression of PECAM-1 and VEGF, but no significant difference was found in hemodynamic variables between MI and CDDP treatment group. We also found that SG exerted a better effect not only on improving hemodynamic function but also on angiogenesis compared with CDDP at the dose of 28.5 mg/kg.

## 5. Conclusion

In conclusion, this study demonstrated that TCM SG dose-dependently improved cardiac hemodynamic function, reduced myocardial infarction size, and promoted myocardium angiogenesis in myocardial infarction rats by upregulating PECAM-1/CD31 and VEGF expression.

## Figures and Tables

**Table 1:** Cardiac hemodynamic parameters.

Group	HR (bpm)	MAP (mmHg)	LVSP (mmHg)	LVEDP (mmHg)	+*dP/dt*.max (mmHg/s)	−*dP/dt*.max (mmHg/s)	CO (ml/min)
Sham (*n* = 10)	342 ± 19	97.72 ± 6.00	116.83 ± 9.00	2.87 ± 2.91	2724.27 ± 321.84	2600.40 ± 300.88	34.67 ± 3.22
MI (*n* = 10)	393 ± 8	42.75 ± 6.29^*∗*^	89.80 ± 6.76^*∗*^	28.51 ± 5.77^*∗*^	1331.23 ± 116.49^*∗*^	1319.31 ± 121.05^*∗*^	14.06 ± 1.43^*∗*^
SG-L (*n* = 10)	380 ± 15	60.68 ± 8.62^*∗*^	86.73 ± 5.04^*∗*^	16.27 ± 5.95^*∗*^	1487.90 ± 129.07^*∗*^	1350.77 ± 129.05^*∗*^	17.04 ± 2.59^*∗*^
SG-M (*n* = 9)	363 ± 17	75.38 ± 10.25^*∗*†^	101.70 ± 7.22^*∗*‡^	9.52 ± 6.13^*∗*†^	2057.06 ± 182.27^*∗*†‡^	1985.16 ± 174.86^*∗*†‡^	19.26 ± 1.25^*∗*^
SG-H (*n* = 9)	378 ± 28	78.87 ± 4.01^*∗*†^	107.54 ± 2.18^*∗*†‡^	14.62 ± 7.91^*∗*^	2127.66 ± 129.62^*∗*†‡^	2046.29 ± 114.83^*∗*†‡^	31.24 ± 1.98^*∗*†‡^
DSDW (*n* = 10)	357 ± 23	61.98 ± 6.87^*∗*^	82.99 ± 4.89^*∗*^	13.93 ± 4.44^*∗*^	1358.70 ± 202.39^*∗*^	1311.50 ± 168.73^*∗*^	15.14 ± 1.67^*∗*^

HR, heart rate; MAP, mean arterial pressure; LVSP, left ventricular systolic pressure; LVEDP, left ventricular end-diastolic pressure; +*dP/dt*.max, maximum positive first derivative of left ventricular pressure; −*dP/dt*.max, maximum positive negative first derivative of left ventricular pressure; CO, cardiac output. ^*∗*^*P* < 0.05 versus sham group, ^†^*P* < 0.05 versus MI group, ^‡^*P* < 0.05 versus DSDW group.
